# Mammalian AKT, the Emerging Roles on Mitochondrial Function in Diseases

**DOI:** 10.14336/AD.2021.0729

**Published:** 2022-02-01

**Authors:** Xiaoxian Xie, Ruonan Shu, Chunan Yu, Zhengwei Fu, Zezhi Li

**Affiliations:** ^1^College of Biotechnology and Bioengineering, Zhejiang University of Technology, Hangzhou, China; ^2^Department of Psychiatry, The Affiliated Brain Hospital of Guangzhou Medical University, Guangzhou, China

**Keywords:** AKT, mitochondrial autophagy, energy dynamics/metabolism, disease, pathway signaling

## Abstract

Mitochondrial dysfunction may play a crucial role in various diseases due to its roles in the regulation of energy production and cellular metabolism. Serine/threonine kinase (AKT) is a highly recognized antioxidant, immunomodulatory, anti-proliferation, and endocrine modulatory molecule. Interestingly, increasing studies have revealed that AKT can modulate mitochondria-mediated apoptosis, redox states, dynamic balance, autophagy, and metabolism. AKT thus plays multifaceted roles in mitochondrial function and is involved in the modulation of mitochondria-related diseases. This paper reviews the protective effects of AKT and its potential mechanisms of action in relation to mitochondrial function in various diseases.

## 1. Introduction

Mitochondria have become a hot research topic due to their important roles in the physiological regulation of the body. The function of mitochondria goes far beyond the production of adenosine triphosphate (ATP). Mitochondria are closely related to a series of physiological phenomena and can respond to alterations in signal molecules, thus contributing to the control of both cell proliferation and death [1]. It has been found that mitochondrial proteins, DNA, and even RNA have effects on cell function in more ways than previously thought, and may be also related to disease phenotypes [2]. It is currently believed that mitochondrial dysfunction is closely related to cardiovascular diseases [3] and neurodegenerative diseases, such as Parkinson’s disease [4, 5], Alzheimer’s disease [6, 7], Huntington's disease [8]; metabolic diseases, such as type 2 diabetes [6, 9] and nonalcoholic fatty liver disease [10, 11]; and depressive disorders [12, 13].

Mitochondrial function may be related to the formation of diseases through a variety of mechanisms including the production of oxidative stress, apoptosis, and cell division, fusion, and mitosis [6]. Mitochondria are the main intracellular source of reactive oxygen species (ROS) [14]. Low levels of ROS are produced by mitochondria through the electron transport chain and are required for cell division and signaling transduction. If the mitochondrial electron transport chain is inhibited by certain mutations, the rate of ROS generation can exceed the rate of clearance, thus leading to excessive ROS [15]. The increase in ROS levels further damages the integrity of mitochondria and reduces the fluidity and permeability of the mitochondrial inner membrane [16]. In particular, mitochondrial DNA (mtDNA) is susceptible to excessive ROS damage because this type of DNA generally lacks effective DNA repair mechanisms and the mitochondrial DNA is not shielded from oxidative stress by histones [17, 18]. Previous studies have shown that mtDNA damage may be the initiating factor of self-propagating mitochondrial dysfunction [19, 20].

Protein kinase B, a serine/threonine kinase (AKT), is the main mediator of the downstream effector protein phosphoinositide 3-kinase (PI3K). There are three subtypes of AKT (1-3) in mammals, which are encoded by different genes and share about 85% amino acid sequence similarity [21, 22]. These isoforms are also similar in structure, and all contain an N-terminal regulatory pleckstrin homology domain, which is considered to be the central kinase domain with serine/threonine specificity, and a C-terminal hydrophobic domain [23]. AKT1-3 play different roles in different tissues, and each has tissue-specific expression. Specifically, AKT1 is ubiquitously expressed, AKT2 is expressed exclusively in adipose, liver, and skeletal tissue that are all insulin-responsive, and AKT3 is expressed exclusively in the brain [24]. AKT deficiency affects cell proliferation and differentiation. AKT1 is the main AKT subtype in tissues and cells, and is widely expressed in the membrane, cytoplasm, and nucleus of T24 and UM-UC-3 bladder cancer cells [25]. Historically, AKT1-3 have been shown to have a similar activation mechanism, although a recent study found that phosphorylation of AKT1-3 is differentially activated in human temporal lobe epilepsy with hippocampal sclerosis [26]. AKT coordinates a variety of signals to mediate cell proliferation and survival in response to external stimuli [27]. It is currently believed that AKT is activated by growth factors, insulin, and DNA damage under normal physiological conditions. AKT is the core of many signaling pathways and is usually suppressed in many types of mitochondrial dysfunction, and as such, activation of AKT can maintain the normal function of mitochondria in several disease states [28, 29]. Interestingly, AKT is activated in response to increased mitochondrial respiratory disturbance [30].

Here, we have outlined the function of AKT and its roles in mitochondrial function. This review may provide a new focus for the therapy of mitochondrial function-related diseases.

**Figure 1.**
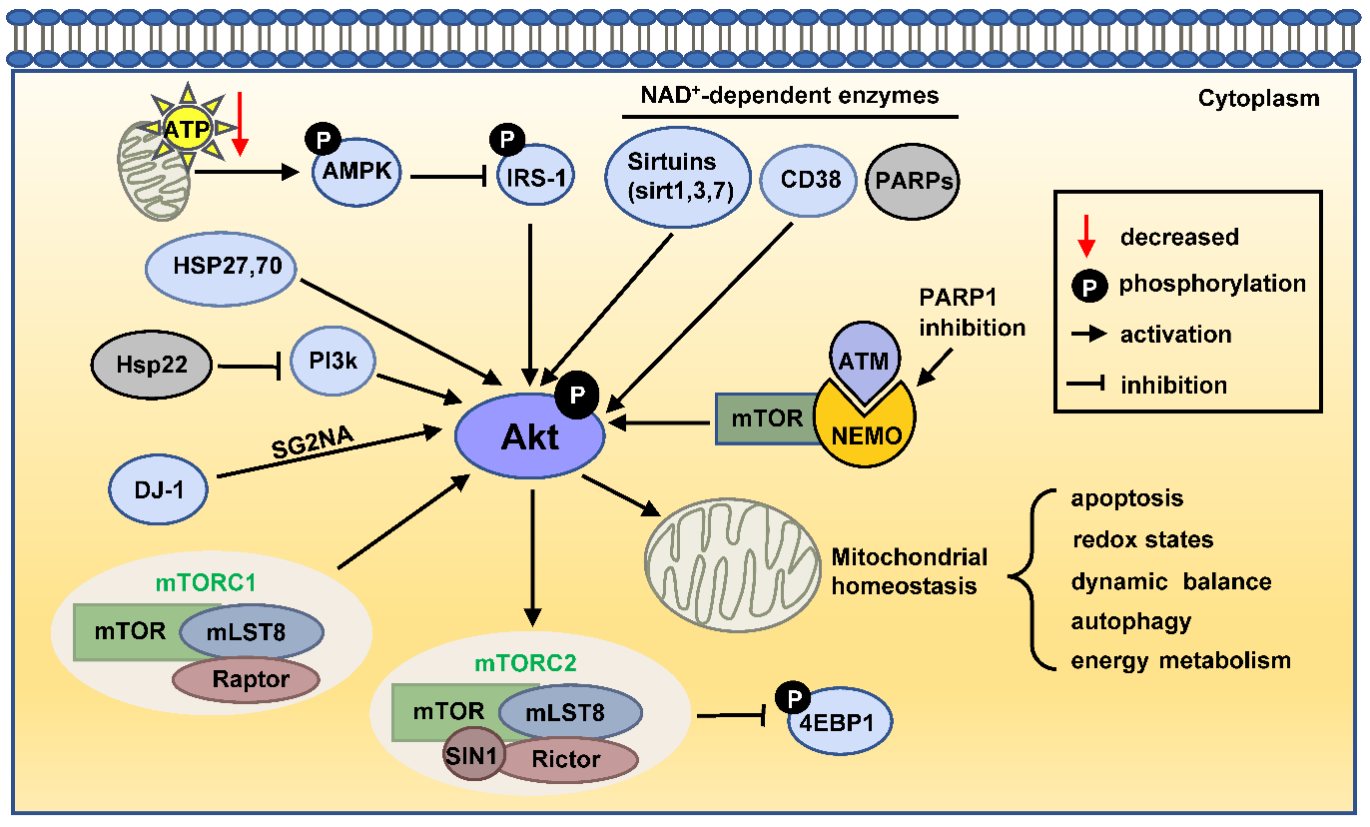
Classification of AKT activations pathways. There are five main types of AKT activation pathways: NAD^+^-related pathways, the DJ-1 pathway, mTOR signaling, the AMPK pathway, and the heat shock protein-related pathway. Abbreviations: PARPs, poly-adenosine diphosphate-ribose polymerases; ATM, ataxia telangiectasia mutated kinase; NEMO, NF-kappa-B essential modulator; mTOR, mammalian target of rapamycin; mLST8, mammalian lethal with SEC13 protein 8; 4EBP1, eukaryotic translation initiation factor 4E binding protein 1; AMPK, adenosine monophosphate-activated protein kinase; IRS-1, insulin receptor substrate-1; PI3K, phosphoinositide 3-kinase; HSP, heat shock protein.

## 2. The signaling pathways activating AKT

AKT pathways are dysregulated in some diseases where mitochondrial dysfunction is present. For example, 1-methyl-4-phenylpyridinium (MPP^+^) induces mitochondrial dysfunction and apoptosis, and is used to mimic Parkinson's disease [31]. In SN4741 cells, MPP^+^ treatment reduced the expression of the active form of AKT, p-AKT (pSer473-AKT) [31]. Therefore, we propose that mitochondrial dysfunction in some diseases is related to the dysregulated AKT pathway. These AKT pathways may be potential therapeutic targets for mitochondria-related diseases. In this review, we summarize several signaling pathways which activate AKT, as shown in Figure 1.

### 2.1. NAD^+^-related pathways

Nicotinamide adenine dinucleotide (NAD)^+^, and its reduced form of nicotinamide adenine dinucleotide (NADH), act as proton acceptors and donors of mitochondria, respectively. A certain ratio of NADH to NAD^+^ is not only a necessary condition for coupling electron transfer chain, but also very important for the production of ATP [32]. NAD^+^ dependent deacetylase (SIRTs) and poly-adenosine diphosphate-ribose polymerases (PARPs) are specific NAD^+^-consuming enzymes that can control the amount of NAD^+^ pools in cells [33]. Previous studies have shown that the regulation of NAD^+^-consuming enzymes affects the function of mitochondria. For example, PARP1 plays the role by modulating the activity of AKT, and inhibition of PARP1 can stimulate the activity of AKT in the absence of oxidative stress [34]. In addition, the inhibition of PARP blocks the PARP1-mediated poly-ADP-ribosylation of ataxia telangiectasia mutated kinase (ATM). In particular, the PARP-dependent interaction between ATM and NF-kappa-B essential modulator (NEMO) forms a ATM-NEMO complex, which leaves the nucleus and binds to mammalian target of rapamycin (mTOR) and AKT in the cytoplasm, resulting in AKT phosphorylation [34]. Furthermore, AKT can be modulated by SIRTs [35-37]. SIRT7, a rarely studied SIRT member, has been found to significantly improve AKT activity [35]. Furthermore, a decrease in SIRT1 protein levels leads to an increase in AKT acetylation levels, which inhibits AKT activity in the hearts of diabetic mice [36]. In contrast, elevated SIRT1 protein levels directly promote the activation of AKT (p-Ser473) [38]. Pyrroloquinoline quinine reduces high glucose-induced human kidney-2 cell apoptosis and oxidative damage by up-regulating SIRT3 and activating the SIRT3/AKT/FoxO3a signaling pathway [37]. Another study showed that SIRT3 is highly expressed in colorectal cancer cells, and loss of SIRT3 leads to the inactivation of AKT, which in turn affects mitochondrial fission and leads to mitochondrial damage. In contrast, the activation of the AKT pathway can counteract mitochondrial fission and promote the migration survival, and growth of colorectal cancer. Elucidating the SIRT3/AKT signaling pathway is of great significance for the study of the mechanism of apoptosis in colorectal cancer [39].

### 2.2. DJ-1 pathway

DJ-1 is encoded by the Parkinson's protein 7 gene, and contains 189 amino acids [40]. The mutation of *DJ-1* gene is the main cause of early, rather than late Parkinson's disease [41]. Endogenous DJ-1 is widely distributed in synaptosomes and membrane organelles such as mitochondria [42]. There is a body of evidence indicating that DJ-1 has antioxidant activity and is critical for maintaining mitochondrial dynamic balance [43]. Increased DJ-1 expression in patients with Parkinson’s disease promotes increased AKT1 protein phosphorylation [44]. Similar findings were found *in vitro* cell experiments. For example, *DJ-1* knockdown can inhibit the AKT signaling pathway and lead to mitochondrial damage [45]. Survival of several kinds of cancer cells is promoted by inhibiting the degradation of DJ-1 and increasing the co-aggregation of DJ-1 and AKT in cells. DJ-1 overexpression can promote the activation of AKT phosphorylation and protect the cells from ROS damage and apoptosis [46]. Previous studies have demonstrated that DJ-1 is recruited to the nucleus and mitochondria under oxidative stress *in vitro* [47]. Further studies found that when cells are in a state of low or moderate oxidative stress, the interaction of DJ-1 and AKT is mediated by S/G2 nuclear autoantigen (SG2NA) [48]. SG2NA increases the co-aggregation of DJ-1 and AKT in cells by inhibiting DJ-1 degradation to promote the survival of several cancer cells [49].

### 2.3. The mTOR signaling pathway

The mTOR signaling is an evolutionarily conserved pathway that controls cell growth and metabolism in response to nutrients, growth factors, and cellular energy levels. There are two types of mTOR complexes with similar structures but completely different functions in mammals, namely mTOR complex 1 (mTORC1) and mTORC2 [50]. The mTOR and mammalian lethal with SEC13 protein 8 (mLST8) are the common components of both mTORC1 and mTORC2. The mTORC1 and mTORC2 regulate each other negatively through the competition for association with mLST8 [51]. The phosphorylation of AKT at Ser473 by mTORC2 leads to its activation [52]. Phosphorylated AKT then activates mTORC1, which subsequently regulates apoptosis, senescence, and catabolism and modulates the expression levels of autophagy-related proteins [53]. Toll-like receptor 4-mediated inflammatory signals enhance the expression of mTORC2-dependent macrophage scavenger receptor by stimulating mTORC2-dependent AKT activation [54]. For example, the mTORC2-AKT-mTORC1 metabolic cascade is involved in the pathogenesis of early atherosclerosis. Taken together, there is a negative regulation between mTORC1 and mTORC2, AKT is a critical activator of mTORC1, and mTORC2 can phosphorylate and activate AKT.

### 2.4 Adenosine monophosphate-activated protein kinase (AMPK) pathway

AMPK, an energy regulator, is induced when cells have reached a certain ATP level, and regulates physiological processes by increasing energy production and inhibiting energy expenditure [55]. Thr172 phosphorylation-activation of AMPK not only increases the expression and translocation of glucose transporters 4 in an insulin-dependent manner, but also stimulates insulin receptor substrate-1 via inhibiting Ser636/639 phosphorylation, to stimulate the activation of PI3K/AKT [56]. AMPK also can inhibit the dephosphorylation of glycogen synthase kinase 3β (GSK3β) catalyzed by AKT [57]. Fumaric acid can activate the AMPK/AKT signaling pathway to inhibit the expression of downstream glucose receptors by consuming neuronal ATP and, in turn, the expression of glucose receptors increase when the AMPK/AKT signaling pathway is inactivated [58]. The above interactions indicate that the AMPK/AKT signaling pathway is invertible and flexible in response to the different physiological needs of cells. The up-regulated ^Thr172^p-AMPK/AMPK and down-regulated ^Ser473^p-AKT/AKT or ^Thr37/45^p-4EBP1/4EBP1 can induce cysteinyl aspartate specific proteinase (caspase)-dependent apoptosis and autophagy [59].

### 2.5. Heat shock protein (HSP)-related pathway

HSPs are cellular chaperone proteins, and act as the key regulators of cellular homeostasis [60], which are closely associated with mitochondrial function [61]. HSPs regulate endoplasmic reticulum-associated degradation, autophagy, mitochondrial protein maintenance, and proteasomes [61]. An increasing number of studies show that AKT can be regulated by some HSPs. For example, HSP22 represses the migration of hepatocellular carcinoma cells by down-regulating the PI3K/AKT signaling pathway, and conversely, knockdown of HSP22 markedly increases the phosphorylation of AKT at Thr308 [62]. The upregulation of HSP70 is associated with the phosphorylation of AKT at Ser473 and contributes to the survival of tumor cell in multiple myeloma[63]. Hsp27 is associated with the activation of AKT via the signal transduction pathway; as the interaction degree between Hsp27 and AKT increases with the level of AKT activity[64]. Additionally, simvastatin promotes HSP27 expression to stimulate the activation of AKT to improve the survival of retinal ganglion cells [65]. The 17-Dimethylaminoethylamino-17-demethoxygeldanamycin, an inhibitor of Hsp90, can enhance apoptosis by inhibiting AKT in the SK-MEL-2 human melanoma cell line under high temperature conditions [66]. Together, these results provide a new insight that distinct HSPs determine cellular survival by regulating the activity of AKT.

**Table 1:** Drugs available targeting AKT.

Drugs	Manipulation of AKT Activity	Outcome	Refs
DIM	Decreased	activates the mitochondrial pathway in malignant melanoma cells, suppresses the proliferation of cells and induces cell apoptosis.	[121]
AS-IV	Increased	relieves ischemic-induced myocardial apoptosis, alleviates myocardium impairment, promotes angiogenesis.	[140]
NBP	Increased	attenuates social deficits and anxiety-like behavior.	[164]
THSG	Increased	improves the behavioral performances of depressive-like mice.	[165]
NR	Increased	ameliorates alcohol-induced depressive behaviours.	[167]
Vanillic acid	Increased	demonstrates antidepressant effects by reducing behavioral despair in the FST.	[168]
Li^+^, VPA, and CBZ	Increased	increases glycogen content in astrocytes, which may be responsible for therapeutic effects of these drugs.	[181]

Abbreviations: DIM, 3,3′-diindolylmethane; AS-IV, Astragaloside IV; NBP, Dl-3-n-butylphthalide; THSG, 2,3,5,4′-tetrahydroxystilbene-2-O-β-d-glucoside; NR, Nicotinamide riboside; Li^+^, lithium salts; VPA, valproic acid; CBZ, carbamazepine; FST, forced swim test; Refs, References.

## 3. The mechanism of AKT regulation in cellular mitochondria

### 3.1 AKT regulates mitochondria-mediated cellular apoptosis

Mitochondria are thought to play an important role in apoptotic events [1]. One of the main pathways of cell apoptosis in mammals is the mitochondrial-dependent pathway, also known as the endogenous pathway, which can alter the permeability of the mitochondrial outer membrane. B-cell lymphoma-2 (Bcl-2) protein is an anti-apoptotic protein located on the mitochondrial outer membrane, which can interfere with programmed cell death. It has now been widely accepted that mitochondria regulate the process of apoptosis by altering the permeability of the mitochondrial outer membrane via regulation of pro-apoptotic proteins [67]. AKT is shown to directly and indirectly regulate apoptosis [68], and to directly regulate cell death through phosphorylation of the pro-apoptotic molecules Bad and Bax, and via interactions with cell death participants [1]. Docosahexaenoic acid induces the mitochondrial-dependent apoptosis by inhibiting the signaling pathways of cAMP response element binding/AKT and extracellular regulatory protein kinase. Specifically, docosahexaenoic acid induces apoptosis in adipocytes by regulating the Bcl-2 protein family members, and, therefore, these proteins may be key checkpoints in the mitochondrial apoptotic pathway [69]. The PI3K-AKT pathway was found to be involved in the apoptosis of A549 cells via experiments using the AKT inhibitor, LY294002. Specifically, after treatment with the AKT inhibitor, LY294002, the expression of p-AKT is decreased, while the pro-apoptotic protein, Bim, is increased in the cytoplasm, which then combines with Bax and is translocated into mitochondria after receiving death signals [70].

In addition, the indirect regulation of apoptosis by AKT is performed by altering the transcriptional level of pro-apoptotic or anti-apoptotic molecules. AKT phosphorylates forkhead box O (FoxO) to interfere with its nuclear transcriptional functions to improve cell survival [71]. Tumor necrosis factor (TNF)-related apoptosis-inducing ligand inhibits the PI3K/AKT/FoxO signaling pathway and localizes FoxO protein from the cytosol to the nucleus in activated hepatic stellate cells, which down-regulates cellular-FLICE inhibitory protein, and subsequently activates apoptosis-related signaling molecules [72]. Decreased levels of phosphorylated AKT induce nuclear accumulation of FoxO1 and up-regulates the FoxO’s target Bim to induce mitochondrial apoptotic pathway in human pancreatic cancer cells [73].

### 3.2 AKT regulates redox states

The activation of AKT promotes the stabilization of NF-E2-related nuclear factor (NRF) 2 via the inhibition of GSK3β and accumulation of cyclin-dependent kinase inhibitor 1A, which inhibits the binding of kelch-like ECH-associated protein 1 (KEAP1) to NRF2.The accumulated NRF2 is then translocated to the nucleus, thereby activating several antioxidant genes including those related to glutathione biosynthesis such as glutamine cysteine ligase catalytic subunit, glutamine cysteine ligase modifier subunit, and glutathione oxidase, as well as other enzymes that are able to reduce and use the protein antioxidant thioredoxin to control cellular ROS production [74]. These antioxidant systems work in both the cytoplasm and mitochondria. The interaction of redox sensors and metabolic sensors improves the ability to assess redox changes [75]. In addition, AKT3 has been reported to up-regulate ROS and it is considered to be the most robust inducer of ROS in isogenic cell lines that express different AKT isoforms. In particular, the activation of nicotinamide adenine dinucleotide phosphate (NADPH) oxidase by phosphorylation of the NADPH oxidase subunit p47^phox^ resulted in the production of ROS induced by AKT3 [76].

AKT is known to regulate redox conditions via other mechanism as well. AKT activation stimulates the synthesis of NADP^+^ by NAD kinase through mono-phosphorylation of Ser44, Ser46, and Ser48 or via the phosphorylation of both Ser44 and Ser46 [77]. In turn, NADP^+^ produces a reduced form of NADPH, which is a major cofactor for reductive metabolism in cells. NADPH can not only fight against oxidative stress, but also participates in the synthesis of essential substances such as fatty acids and deoxynucleotides associated with daily life activities. In short, AKT mediates a series of mammalian cellular reactions to promote cellular adaptation to changes in the environment by regulating the redox state [78].

### 3.3 AKT regulates the dynamic balance of mitochondria

The dynamic balance of mitochondria depends on the balance of fission and fusion, which directly affects the function of mitochondria. Mammalian mitochondrial fission is mainly regulated by dynamin-related protein 1 (Drp1), Fission 1 (Fis1), and dynamin-2, while the fusion of mitochondria is controlled by several proteins including mitofusin 1 (Mfn1), Mfn2, and Optic Atrophy 1 (OPA1) [79]. Under normal physiological conditions, mitochondria are interconnected by tubular networks. Mitochondrial fragmentation is induced when the cells are exposed to stress conditions, such as an imbalance of intracellular Ca2^+^ homeostasis, consumption of ATP pools, and stimulation by environmental pollutants. The fragmentation of mitochondria under stress conditions is observed as a pro-survival response [80]. There is an evidence that AKT regulates mitochondrial fusion and fission [38, 81]. Activation of the AKT signaling pathway by melatonin treatment attenuated TNF-α-stimulated disruption of mitochondrial dynamics in the hepatocytes [82]. The mechanism by which AKT protects the heart from ischemia-reperfusion injury may be meditated by AKT regulation of Mfn1 to induce mitochondrial elongation [83]. In addition, the physiological levels of Mfn2 expression are observed to be strongly related to the AKT signaling pathway [84]. Also, activation of AKT increases the expression of OPA1 induced by insulin to promote mitochondrial fusion. Similarly, AKT activation can regulate mitochondrial fission by mediating the activity of Drp1. In contrast, supplement of AKT inhibitor CB-124005 blocked the translocation of Drp1 from the cytosol to mitochondria, and thus led to excess ROS generation and mitochondrial fission [38, 81]. The levels of phosphorylated AKT and Fis1 are negatively correlated, and indicate that the AKT pathway is involved in Fis1-mediated mitochondrial fission [85]. Taken together, we hypothesize that AKT regulates mitochondrial dynamics by regulating the expression of mitochondrial fusion and fission-related proteins.

### 3.4. AKT regulates autophagy

Autophagy is a dynamic recycling system that removes and degrades the waste product in cells and generates new energy for cellular renovation [86]. Defects in autophagy in physiologically senescent satellite cells have been shown to lead to elevated mitochondrial dysfunction [87], whereas re-establishment of autophagy rescues mitochondrial dysfunction [88], suggesting that the regulation of autophagy is a potential target for mitochondrial dysfunction.

#### 3.4.1 AKT, in combination with PI3K and mTOR, regulates autophagy

Autophagy is a negative feedback loop in response to nutrient deficiency. This deficiency can induce cells to initiate autophagy, where the proteasome degrades ubiquitinated substrates to produce amino acids to combat starvation conditions, which is considered as a beneficial event. Then, the restoration of amino acid levels activates mTORC1, and autophagy is reduced, thus preventing excessive autophagy. Growing evidence has suggested that PI3K/AKT/mTOR pathway not only mediates anti-apoptotic signaling, but may also participate in the modulation of autophagy via the ubiquitin-proteasome system in mammalian cells. Naringin, a lipid-lowering drug, is found to relieve autophagy by activating the PI3K/AKT/mTOR pathway and inhibiting the level of cellular autophagy-related protein Beclin 1 [89]. Another study showed that the possible mechanism was related to myocardial autophagy acceleration through the inhibition of the AKT/mTOR pathway, whereby valproic acid attenuates sepsis-induced myocardial dysfunction [90]. Similarly, caffeine is found to enhance autophagy by inhibiting PI3K/AKT/mTOR/p70S6K signaling pathway, eventually inducing apoptosis [91].

Mechanistically, AKT is a critical activator of mTORC1, and this activation of mTOR pathway leads to the inhibition of autophagy via Unc-51-like kinases 1/2 [92]. The formation of autophagosomes is essential for mitochondrial autophagy. After the microtubule-associated protein light chain 3 (LC3) is converted into its lipidation form LC3-II and transferred to the autophagosome membrane, the autophagosome then fuses with the lysosome to complete the autophagy process [92]. The down-regulation of phosphorylation of AKT/mTOR and AKT/FoxO3a signal pathways and the up-regulation of phosphorylated AMPK have been shown to regulate autophagy and mitochondrial quality control [93]. From the above examples, it can be concluded that the activation of AKT regulates the termination of autophagy.

#### 3.4.2 AKT regulates Pink1/Parkin-mediated mitophagy

Mitochondrial quality control is necessary to maintain normal mitochondrial function. There are various self-repair mechanisms in mitochondria. When mitochondria are damaged and cannot be repaired, mitophagy, a specialized autophagy pathway, leads to the degradation of mitochondria in lysosomes [94]. Two Parkinson’s disease-linked proteins are the serine/threonine protein kinase Pink1 and the E3 ubiquitin ligase Parkin [95]. When mitochondria membrane potential drops, Pink1 accumulates on the damaged mitochondrial outer membrane via forming a large complex with TOM complex and undergoes intermolecular auto-phosphorylation at residues S228 and S402, leading to its activation. After being activated by Pink1, Parkin accumulates via translocating from the cytoplasm to mitochondria, resulting in the ubiquitination of the outer membrane proteins of mitochondria, which have more than 2,000 types including Mfn1/2 and TOM20, and activation of autophagy [96]. The Bcl-2 family protein Bcl-xL not only directly interacts with Parkin in the cytoplasm to prevent its aggregation into mitochondria to bind Pink1, but also directly interacts with Pink1 to inhibit Pink1/Parkin-mediated mitochondrial phagocytosis [97]. Furthermore, the Pink1/Parkin-mediated mitochondrial phagocytosis pathway plays an important role in neuroprotection [98]. Interestingly, AKT can regulate mitochondrial selective autophagy [99]. Defects in the PI3K/AKT signaling pathway and mitochondrial autophagy are important causes of mitochondrial dysfunction caused by I/R [100]. Electroacupuncture treatment ameliorates nitro/oxidative stress-induced mitochondrial functional damage and clears mitochondria by Pink1/Parkin-mediated mitochondrial autophagy via the AKT pathway [100]. Together, the data suggest that AKT regulates mitochondrial autophagy by regulating mTOR and Pink1, which are key downstream targets of the AKT signaling pathway.

### 3.5. AKT modulates energy metabolism

**Figure 2.**
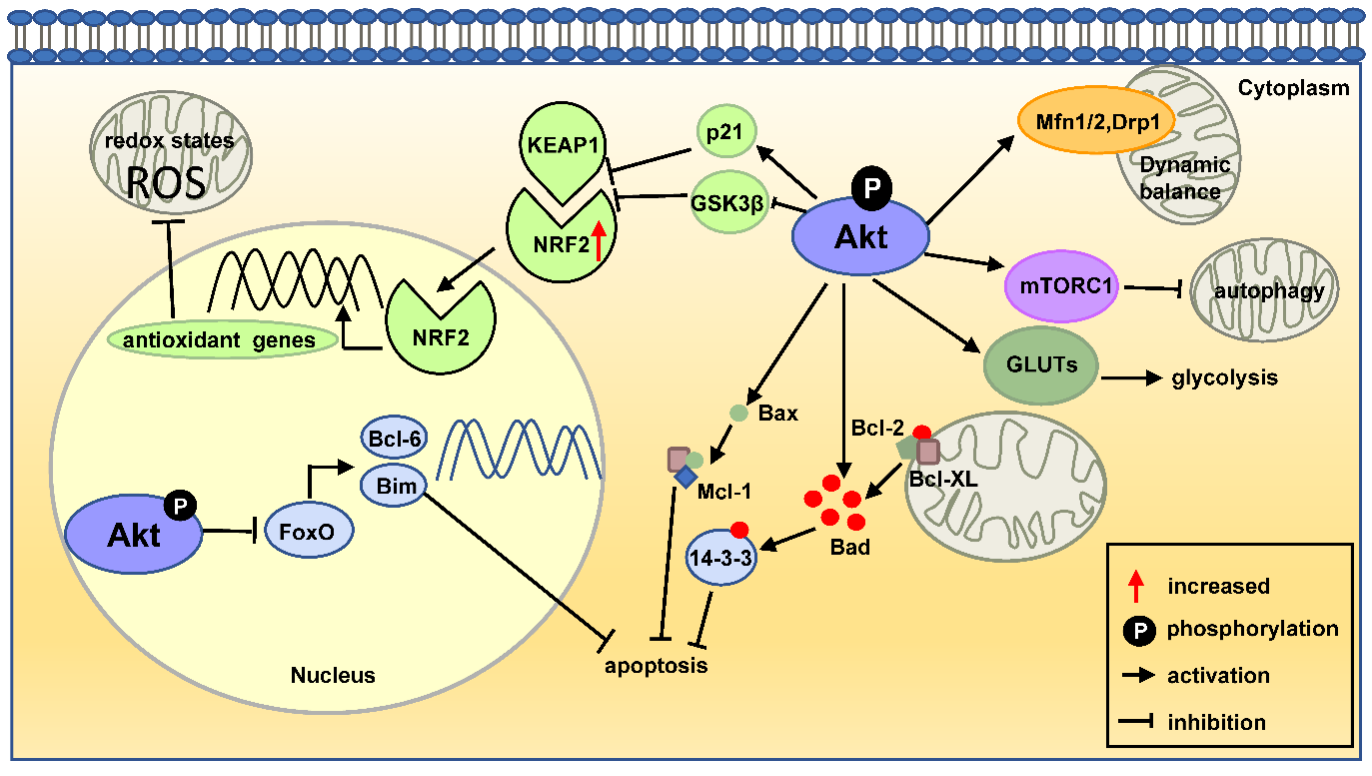
The mechanism of AKT regulation in cellular mitochondria. AKT regulates apoptosis, redox state, dynamic balance, autophagy, and energy metabolism. Abbreviations: Bcl-2, B-cell lymphoma-2; FoxO, forkhead box O; NRF2, NF-E2-related nuclear factor 2; KEAP1, kelch-like ECH-associated protein 1; GSK3β, glycogen synthase kinase-3β. GLUTs, glucose transporters.

Energy metabolism is one of the most basic characteristics of life and is highly organized within cells [101]. Numerous studies have shown that AKT activation can regulate mitochondrial-dependent energy metabolism [102]. For example, double knockout of AKT2 and AMPK exacerbates high fat diet-associated loss in mitochondrial biogenesis [103], and lack of AKT2 in mice is sufficient to produce high insulin levels and diabetic phenotypes [104, 105]. The PI3K/AKT signaling pathway can regulate glucose uptake and glycolysis in adherent adult stem cells by regulating the expression of the glucose transporter, while glycolysis provides intermediates for the synthesis of many metabolites [106]. Similarly, the activation of the PI3K/AKT signaling pathway in HepG2 cells significantly increases the protein expression levels of the rate-limiting enzymes hexokinase2 and phosphofructokinase, which control the glycolysis process [107]. The activated glycolysis induced by AKT enhances its coupling with oxidative phosphorylation, which indirectly promotes oxidative phosphorylation [108]. Increasing evidence has shown that the decreased activity of AKT kinase is accompanied by impaired glucose transport and metabolic defects in insulin-sensitive tissues, such as skeletal muscle [109-111]. In addition, insulin rapidly regulates energy response through the PI3K/AKT pathway to maintain mitochondrial respiration in human embryonic stem cells [112]. Multiple studies have shown that FoxO1 factor can also regulate insulin signal transduction and glucose metabolism. In addition, there is a feedback inhibition between FoxO1 and AKT, and changes in the activity of FoxO1 alter AKT activation to impair glucose metabolism [113-115]. Together, AKT is necessary for glucose uptake to maintain glucose homeostasis, which in turn regulates glycolytic and oxidative metabolism. As presented in Figure 2, the mechanism associated with AKT modulation of phenotypes in cellular mitochondria is summarized, including mitochondria-mediated cellular apoptosis, redox states and the dynamic balance among mitochondria, autophagy, and energy metabolism.

## 4. The roles of AKT in mitochondria-related diseases

### 4.1. AKT and cancer

Changes in the AKT pathway have been observed in many human malignancies, and constitutively active AKT is sufficient to induce oncogenic transformation of cells and tumor formation. AKT can regulate a wide range of downstream targets to mediate tumor-associated cell processes including cell growth, cell cycle progression, survival, migration, and conversely, inhibition of AKT signaling, leading to apoptosis and cell proliferation of tumor cell with over-elevated AKT activity [116]. Notably, AKT2 is the main regulator that promotes a higher survival rate of breast cancer cells under hypoxia because hypoxia induces the expression of AKT2, but not AKT1 or AKT3 [117]. Furthermore, a report showed that numerous components of the PI3K/AKT pathway were found to be more frequently targeted in the processes of amplification, mutation, and translocation in cancer patients with abundant activation of the pathway, indicating the potential to exploit the PI3K/AKT pathway for cancer drug discovery [118].

Mutations in apoptotic regulators may be coupled with the occurrence of tumorigenesis [119]. Indeed, evasion of apoptosis is recognized as a hallmark of cancer. AKT is considered as an apoptotic regulator and is activated in many cancers. Rapamycin reverses the chemoresistance in AKT-overexpressing lymphomas in a murine lymphoma model via disruption of the AKT/mTOR/eIF4E signaling pathway by inhibiting mTOR activity. These observations show that AKT signaling, in combination with mTOR and eIF4E, is an important mechanism of modulating oncogenesis and drug resistance [120]. Similarly, association with pro-apoptotic phosphatase and tensin homolog deleted on chromosome ten (PTEN)/AKT signaling is essential to the antitumor effects of 3,30-diindolylmethane (DIM), which can modulate the expression of proteins in cancer-related pathways. Specifically, DIM can elevate the expression of PTEN, which then inhibits AKT phosphorylation at Thr308, and coupled with downstream signaling that inhibits proliferation, induces apoptosis of A375, Mel501, and SKMel28 cells, but not A2058 cells (Table 1) [121]. This inhibitory response was also observed in *Akt3*-overexpression cells, and the proliferation of triple AKT knockout lung fibroblasts and kidney-derived cells was rescued by AKT3, although the proliferation was lower than in cells rescued by AKT1 or AKT2 [76]. The slow proliferation of the cells expressing *Akt3* is associated with high expression of *p53* and its direct transcriptional target, miR-34, which is consistent with previous observations showing that the expression of *AKT3* was positively associated with the levels of p53 in a variety of human tumors [76]. Similar effects were also observed in malignant melanoma cells, in which PTEN and inactivation of AKT signaling were involved in DIM-mediated proliferation-inhibition and apoptosis-induced effects. PTEN/AKT signaling decreases the expression of cytosolic AIF, Cyt C, cleaved caspase-3 and -9, Bax, and Bid, and also enhances the expression of Bcl-2 and the anti-apoptotic/pro-apoptotic protein ratio, leading to mitochondria-mediated apoptosis [121]. After combined treatment of carbon ions radiation and tigecycline, increased phosphorylated AKT and decreased phosphorylated AMPK were observed. These two proteins antagonistically target phosphorylated mTOR to modulate mitochondrial translation proteins and caspase 9, which affected autophagy and apoptosis, thus inhibiting the proliferation of lung cancer cells [122].

Dysregulation of AKT is an important cause of many human cancers [123]. Previous studies have shown that AKT kinase activity is up-regulated by about 40% in breast, epithelial ovarian, prostate, and gastric cancers [124]. Other studies have revealed that increased oxidative stress and changes in cell metabolism may lead to tumor initiation and/or progression [125]. Increased oxidative stress has an impact on genomic stability and increases the frequency of mutation [126]. Given that mitochondria are the main source of cellular energy and ROS generation, AKT may be closely related to energy metabolism and redox state in cancer. Furthermore, metabolic changes that promote anabolic growth may also support the growth of tumors [127]. Therefore, inhibiting the AKT pathway, including AKT itself, and its upstream regulators and downstream effectors may become an effective molecular target for cancer treatment [116].

### 4.2. AKT and cardiovascular disease

Due to unhealthy diet or genetic factors, cardiovascular diseases are increasing worldwide [128]. Common cardiovascular diseases include coronary atherosclerosis, hypertension, and myocarditis. They can develop into congestive heart failure, which can result in death in severe cases [129]. AKT, as a central mediator in cardiomyocyte signaling, has a protective effect in cardiovascular disease [130-133]. For instance, AKT activation can increase cardiac angiogenesis and promote a reversion of metabolism in postnatal life to a fetal phenotype [134]. AKT1 phosphorylation and its nuclear translocation contribute to maintain physiological function in cardiomyocytes [135]. AKT3 maintains mitochondrial homeostasis through inhibition of PGC-1α nuclear localization and is also required for angiogenesis [136]. Deficiency of AKT3 promotes atherosclerosis in mice [137], which to some extent supports the fact that morphological changes of mitochondria and their dysfunction are closely related to cardiovascular disease [138, 139]. Additionally, the activation of the PTEN/PI3K/AKT signaling pathway can stimulate Astragalus membranaceus astragaloside IV, which is a major monomer extracted from a classic Chinese herbal medicine, to promote angiogenesis after myocardial infarction (Table 1) [140].

Among the three isoforms, AKT1 is most closely related to cardiovascular disease because AKT1, not AKT2, is critical for ischemic and vascular endothelial growth factor-mediated angiogenesis [141]. However, long-term AKT activation has also been observed in many cardiovascular diseases. In high-fat diet mice, long-term activation of AKT1 induced increased vascular senescence and vascular dysfunction. In contrast, deficiency of AKT1 leads to resistance to vascular senescence [142], whereas increased AKT1 activity induces cardiac hypertrophy [143]. In addition, sustained AKT activation induces pathological cardiac hypertrophy, impaired coronary angiogenesis, and cardiac dysfunction [144]. AKT2 ablation protects against cardiac aging by restoring mitochondrial integrity [145], and may protect against paraquat toxicity-induced cardiac contractile defects through regulation of Nrf2 activation and mitochondrial homeostasis [146]. Therefore, AKT must maintain a proper balance between the level of activation and duration, as long-term activation of AKT will eventually produce adverse consequences, and even heart failure [147].

### 4.3. AKT and neurodegenerative disease

Neurons contain a large number of mitochondria, and mitochondrial homeostasis is one of the necessary conditions for neuronal homeostasis [148]. Dysfunctional mitochondria produce less ATP and an increased level of ROS, which can trigger neuronal stress and contribute to neurodegeneration [148]. There is evidence that protein Grb10-interacting GYF protein 2 (GIGYF2), a protein associated with Parkinson’s disease, has four typical AKT consensus site motifs, which can modulate AKT signaling, although it is not clear whether GIGYF2 is a substrate of AKT. As a key signaling pathway, AKT is shown to regulate survival and synaptic plasticity in neurons [149, 150], and is necessary for the protective effect of brain-derived neurotrophic factor in neurodegenerative disease [151]. In addition, activation of the AKT pathway is involved in the mechanism of human platelet lysates-mediated prevention of neuron loss associated with neurodegenerative diseases; an observation similar to that found in in vitro models of Parkinson’s disease [152]. Direct pharmacological AKT activation rescues Alzheimer’s-like memory impairments and aberrant synaptic plasticity [153]. In addition, many neurodegenerative diseases, such as Parkinson's disease, are thought to occur when misfolded proteins are not degraded in timely manner through autophagy [154]. A previous study implies that the disruption of autophagy is correlated with neurodegenerative disorders [155]. Indeed, the hyperactivation of the PI3K/AKT/mTOR pathway induced autophagy disruption, leading to disrupted clearance of amyloid β peptide and tau, synaptic loss, and cognitive decline in Alzheimer's disease patients [156]. miR-181b can regulate autophagy in a model of Parkinson’s disease by targeting the PTEN/AKT/mTOR signaling pathway [157]. Although neuronal autophagy appears to be a protective process in the nervous system, pathogenic autophagy associated with neuronal death does occur [158]. Thus, a better identification of autophagic stress and PI3K/AKT/mTOR signaling pathway activation for the treatment of neurodegenerative diseases are needed [159].

### 4.4. AKT and psychiatric disorders

Accumulating evidence has shown that mitochondria, as a key organelle for energy metabolism in the body, are closely related with psychiatric disorders, such as major depression, bipolar disorder, and schizophrenia [160, 161]. As a key node of the cellular energy metabolism pathway, AKT plays an important role in mitochondrial energy metabolism. The antidepressant drugs are listed in Table 1.

#### 4.4.1 Major depression

Evidence has shown that the pathology of major depression may be alleviated by brain intracellular signal transduction systems [162]. Activation of AKT ameliorates the effects of chronic social defeat, which is causally related to major depression [163]. In a previous study, the compound, Dl-3-n-butylphthalide, attenuated mouse behavioral deficits via activation of the AKT signaling pathway in the hippocampus of chronic social defeat stress-induced depressive mice [164], and a similar phenomenon was observed in the prefrontal cortex of chronic-restraint stress induced depression-like mice [165]. Also, SIRT6 overexpression in hippocampal neurons induced depressive behaviors via inhibition of the AKT signaling pathway in depressed rodents [166]. Moreover, many antidepressants can mediate depression-like behavior via activation of the AKT pathway, for example, by activating the AKT/ GSK3β /β-catenin signaling pathway in the hippocampus to attenuate alcohol-induced depression in mice. Nicotinamide riboside, a precursor of NAD^+^, can activate AKT activity to reduce depression-like behaviors [167], similar to the antidepressant vanillic acid [168]. In addition, many studies have shown that except for exogenous antidepressants, exercise therapy is an effective treatment for depression. Physical exercise can mediate the proliferation and differentiation of neural stem cells via the AKT pathway, and thus act as an antidepressant to enhance the integrity of hippocampal structure and function [169]. Together, the data indicate that a significant correlation between AKT activity and depression is established, and AKT may become a new target for the treatment of mood disorders.

#### 4.4.2 Bipolar disorder

Strong evidence supports that mitochondrial dysfunction plays a key factor in the pathogenesis and pathophysiology of bipolar disorder [170]. Mitochondrial dysfunction affects the release of neurotransmitters, including monoamines and glutamate, which are thought to be related to bipolar disorder [171, 172]. Dopamine is a catecholamine neurotransmitter. Brain dopamine receptors have been regarded as targets for compounds that were developed for the treatment of bipolar disorder [173]. Several lines of evidence have shown that dopamine receptors can exert their biological effects through AKT signaling pathways [174, 175]. Therefore, AKT is a downstream signaling effector of the critical mediators of a neurotransmitter system [176, 177]. There is also increasing evidence that the dysfunction of intracellular signaling cascades, including the AKT pathway, is evident in bipolar disorder [178, 179]. For example, decreased AKT1 and mTOR mRNA expression was observed in bipolar disorder patients without medications compared with healthy controls [180]. Furthermore, after lithium treatment, the changes in AKT1 expression were positively associated with improvement of symptoms [178]. Another report demonstrated that the Cav-1/PTEN/PI3K/AKT/GSK-3β pathway was regulated by treatment with three mood stabilizers in primary cultured astrocytes including lithium, valproic acid, and carbamazepine. These three mood stabilizers decreased Cav-1 expression levels and membrane content of PTEN, promoted PI3K and AKT activity, and inhibited GSK-3β activity by increasing its phosphorylation [181].

#### 4.4.3 Schizophrenia

Previous studies of postmortem brains, animal models, and genetic association on schizophrenia showed the dysfunction of the AKT signal pathway may play a critical role in the pathophysiology of schizophrenia [182]. For example, a decrease in AKT1 levels and GSK3β phosphorylation were found in the peripheral lymphocytes and brains of schizophrenia patients [183]. Also, an AKT1 haplotype linked to lower AKT1 protein levels was associated with schizophrenia. The authors also showed that AKT1 deficiency contributed to a greater sensitivity to the sensorimotor gating-disruptive effect of amphetamine. Another report demonstrated that phosphorylated AKT levels were decreased in hilar neurons of the dentate gyrus of postmortem brain tissues of schizophrenia patients [184]. Furthermore, decreased AKT content and activity were detected in the dorsolateral prefrontal cortex of schizophrenia patients compared with controls [185]. PI3K subunit p110δ gene is reported to be associated with schizophrenia. Furthermore, PI3K-AKT-GSK3 pathway is involved in pathophysiology of schizophrenia [186, 187].

### 4.5 AKT and Aging

Recent findings have shown that a potential role of AKT in pathological changes induced by age or aging in the heart, and that suppression of AKT signaling pathway can extend the lifespan in aging individuals [145, 188]. In contrast, chronic AKT activation has been to shown to inhibit autophagy by triggering the downstream signal NF-κB, thus the reducing the life span[ 189]. The above results are consistent with another study that showed that impairment of autophagy can accelerate aging [190]. Similarly, overactivation of mTOR, upstream of AKT signaling, aggravates aging through impaired autophagy [191]. Moreover, cardiac hypertrophy was observed in an aging murine model induced by AKT overexpression, which is considered as a common phenotype of aging hearts [192, 193]. Strikingly, a study showed that exercise training can increase autophagy in aged rats and prevent muscle wasting by downregulating the phosphorylation of AKT and mTOR [194]. In short, the AKT/mTOR pathway is a key regulator of autophagy in aging [195]. We believe that autophagy can be improved by targeting the AKT pathway to improve protein and organelle quality control pathways, which is considered to be an emerging contributor to age-related dysfunction [196].

## 5. Conclusion

In conclusion, mitochondrial dysfunction is the main cause of many diseases, and the loss of AKT is closely associated with the cause of various diseases. Here we have reviewed the important signaling pathways that can modulate the activity of AKT, which in turn impact mitochondria-related diseases. Furthermore, we show that AKT regulates mitochondrial function via several mechanisms, including those associated with apoptosis, autophagy, redox states, dynamic balance, and metabolism. Therefore, AKT may be an important target for the treatment of mitochondria-linked diseases. However, this field is still in its initial phases, and a great deal of work needs to be performed before determining whether the activation or inhibition of AKT modulates disease progression.
